# Relationship of Lifespan Physical Activity Engagement With Quality of Life in Community‐Dwelling Adults: A Retrospective Study

**DOI:** 10.1002/hsr2.71153

**Published:** 2025-08-06

**Authors:** Ewerton P. Antunes, Wagner L. Prado, Daniel Canhin, Claudiele C. M. Silva, Luis Alberto Gobbo, Gerson Ferrari, Jorge Mota, William R. Tebar, Diego G. D. Christofaro

**Affiliations:** ^1^ School of Technology and Sciences São Paulo State University (Unesp) Presidente Prudente São Paulo Brazil; ^2^ California State University San Bernardino San Bernardino California USA; ^3^ Núcleo de Investigación en Ciencias del Movimiento Universidad Arturo Prat Chile; ^4^ Facultad de Ciencias de la Salud Universidad Autónoma de Chile Providencia Chile; ^5^ Escuela de Ciencias de la Actividad Física, el Deporte y la Salud Universidad de Santiago de Chile (USACH) Santiago Chile; ^6^ Research Center in Physical Activity Health and Leisure (CIAFEL)‐Faculty of Sports‐University of Porto (FADEUP) and Laboratory for Integrative and Translational Research in Population Health (ITR) Porto Portugal

**Keywords:** adolescence, adulthood, childhood, general health, physical exercise, sports

## Abstract

**Background and Aims:**

Lifelong physical activity (PA) engagement has been linked with several health benefits, such as the prevention of cardiovascular diseases. However, longitudinal studies investigating the association of lifelong PA with quality of life (QoL) needs to be more explored in literature.

**Methods:**

We analyze the association of lifelong PA engagement with quality of life (QoL) ini = 842 community‐dwelling adults (56.5 ± 18.3 years, 61.7% of women). PA engagement during childhood and adolescence was retrospectively assessed considering supervised sports practice outside the school for at least 1 year. Current PA was assessed by Baecke score, and classified as highly active (4th quartile) and less active (1st–3rd quartile). QoL were assessed through by the SF‐36, and classified as high (4th quartile) and low (1st–3rd quartile) QoL for each domain. Multiple logistic regression models (adjusted by sex, age, socioeconomic status, and disabling chronic conditions) analyzed the association between lifelong PA engagement with QoL.

**Results:**

Participants who engaged in PA in childhood and those currently classified into the highest quartile of Baecke score were more likely to present high QoL in isolated statistical models. Specific combinations of PA engagement in childhood + adolescence, childhood + adulthood, and adolescence + adulthood were associated with high physical functioning (OR: 3.34; 3.39; and 2.82), vitality (OR: 2.63; 2.08; and 2.40). Clustering life stages of PA engagement showed an incremental association for high physical functioning (2 stages OR: 2.49; 3 stages OR: 5.53), general health status (2 stages OR: 2.09; 3 stages OR: 2.79), and mental health (1 stage OR: 1.42; 3 stages OR: 2.79).

**Conclusion:**

Lifelong PA engagement is positively associated with QoL in adults. Actions for QoL promotion should focus on adherence of PA practice at all life stages, but this specific sample profile limited the generalization of results.

## 1 Introduction

Childhood and adolescence are characterized by physical, social, and behavioral changes, and such habits may be consolidated in adulthood, as physical activity engagement (PA) [1]. Adults who practiced PA during childhood and adolescence showed lower body adiposity and lower inflammatory profile [2], better cardiac autonomic modulation [3], lower odds of having cardiovascular risk factors [4], lower mentally passive sedentary behavior [5], and higher levels of vigorous PA [6]. Besides cardiometabolic benefits, PA can also promote mental health benefits, including improvement in the quality of life (QoL) [7].

QoL refers to self‐report of functioning and well‐being in different life domains, being a subjective parameter related to the perception of life experiences, including functioning and well‐being in mental and physical health, social aspects, and general living conditions. Due to its holistic nature, QoL is typically assessed using validated multidimensional instruments, such as the Short‐form Health Survey‐36 (SF‐36), which evaluates and scores QoL across eight dimensions in a quantitative way [8]. The QoL is a critical outcome variable in PA research, since it can be differently influenced by PA across its dimensions of health, in a person‐centered perspective.

The regular engagement in PA is associated with improvements in the physical, mental, and social aspects. In the physical domain, the benefits of PA address cardiovascular health [9], strengthening muscles and bones [10], as well as body weight management [11] and chronic diseases prevention [12], contributing to self‐efficacy in daily tasks. Regarding mental health, PA practice promotes the release of pleasurable substances such as endorphins and serotonin [13, 14], which could contribute to general well‐being. Additionally, PA engagement can be associated with improvement in social bonds and sense of belonging [15]. These benefits of PA can interplay in such a way that improvement in one aspect can enhance the others and, consequently, increase the global QoL level.

The benefits of PA practice in QoL were demonstrated in various populations [7] and across different stages of life [16, 17, 18, 19]. However, it is not established whether lifelong PA engagement would be associated with better QoL, since previous studies did not consider a retrospective approach. Investigating PA engagement at different life stages is essential because lifestyle habits may change over time, influenced by biological, social, and environmental factors [20]. In addition, continued PA engagement is associated with long‐term benefits such as improved physical functionality, lower risk of chronic diseases, and improved mental health [21]. Moreover, studies with larger household randomized samples are needed to investigate lifelong PA engagement and QoL in an epidemiological spectrum. Therefore, this study aimed to analyze the association of lifelong PA engagement with QoL in adults, considering the periods of childhood, adolescence, and adulthood in a household‐based, randomly selected sample of community‐dwelling adults.

## 2 Methods

This is a retrospective observational study described according to the Strengthening the Reporting of Observational Studies in Epidemiology (STROBE) Guidelines.

### 2.1 Sample

Eight hundred and forty‐two adults aged 18 years and older (519 being women) participated in this study. The study was carried out in the city of Presidente Prudente, located in the Southeastern region of Brazil, with a population of 207,610 inhabitants, of whom 176,124 were adults aged ≥ 18 years old. For the study purpose, a survey was carried out by stratifying all the streets according to the neighborhood, postal code, and geographic location into five geographical regions (North, South, East, West, and Central). Approximately 3000 streets were identified in the city, and randomized lists were created within each geographic region using an online randomization tool—http://www.random.org/lists—from which the planning and schedule for data collection were developed. In each of the selected streets, all existing households were visited, with a call, explanation about the research, and an invitation to participate extended to those who met the inclusion criteria and agreed to participate in this study. The randomization of the streets occurred according to the number of individuals interviewed, so that as many streets were drawn as were necessary to obtain a minimum sample in each region [22, 23, 24].

The minimum sample size was calculated considering the following parameters: a population of ~176,000 inhabitants aged 18 years and more; an outcome rate of 50% as considered in epidemiological studies with unknown prevalence or multiple outcomes [25]; a tolerable error up to 5% and 95% confidence levels; being applied a design effect correction of 1.5 due to the conglomerate sampling process. Therefore, a minimum of 576 individuals were required for the present study sample.

To be eligible to participate, the participants needed to be 18 years old or older, be residents in the city for at least 2 years, not have any disability in understanding and answering the questionnaires, as well as some functional barriers that made standing difficult. People with mobility difficulties, using walking sticks, or who were bedridden were not eligible for the study. Those who did not correctly answer the questionnaires or participate in the anthropometric measurements were excluded from the sample. All participants signed the informed consent form, and the study was previously approved by the institutional committee of ethics in research by São Paulo State University (CAAE protocol 45486415.4.0000.5402‐06.19.2015; approved on June 23, 2015). Data collection was carried out in 2016 and 2017.

### 2.2 Quality of Life

QoL was assessed by the 36‐Item Short Form Health Survey (SF‐36), which provides QoL scores in eight different domains: physical functioning, role physical, bodily pain, general health, vitality, social functioning, role emotional, and mental health [26]. Total score ranges from 0 to 100, whereas 0 indicates the worst and 100 the best QoL score. The sample was divided into quartiles, since the use of 25th, 50th, and 75th percentile cutoffs has been recommended by the SF‐36 developers as a more informative method for the distribution of scores [27], and those individuals located in the highest quartile (Q4) were classified as high QoL, while those located in the lowest quartiles (Q1–Q3) were classified as low QoL.

### 2.3 Early Physical Activity Engagement

The PA engagement in childhood and adolescence was assessed by means of sport practice by two questions: “When you were between 7 and 10 years old, did you practice any type of organized/supervised sport outside the school for at least 1 year?”, and “When you were between 11 and 17 years old, did you practice any organized/supervised sport outside the school for at least 1 year?”. Affirmative responses to one or both the two questions were considered to classify the respondents as PA engagement at youth. These questions were adopted due to the ease of understanding and for having shown good reliability, and have been used in several previously published epidemiological studies [3, 5, 6, 28, 29].

### 2.4 Current Engagement in Physical Activity

The current practice of physical activity was assessed by the Baecke questionnaire [30], which evaluates PA in three different domains: work/occupational, sports practice, and leisure time/commuting PA. This instrument provides a dimensionless domain score, and the sum of all domains provides a total PA score. This instrument is validated for use in the Brazilian adult population [31]. Participants in the highest quartile of the total physical activity score (Q4) were classified as being “highly active”. Participants located in quartiles Q1, Q2, or Q3 were classified as less active. This cutoff was adopted for showing good reproducibility against objectively measured physical activity at moderate‐to‐vigorous level [31]. The Baecke questionnaire has a specific question regarding the current engagement in sports practice (“Do you or did you practice sports or physical exercise within the past 12 months? Yes/No”) which was also considered to define adulthood sports practice engagement in alignment with the childhood/adolescent PA engagement question, being named as “Adulthood sports engagement”.

### 2.5 Continuity of Physical Activity Across Lifespan

Participants who reported to practice PA in the three life stages (childhood, adolescence, and being highly active in adult life) were classified as “continuous PA”.

### 2.6 Socioeconomic Condition

The Brazilian Economic Classification Criteria [32] were used to assess the socioeconomic status of the sample. This instrument considers the level of education and the number of certain rooms (such as the number of bathrooms in the house) and consumer goods in the home (e.g., car, TV, freezer, housemaid, etc.). In this instrument, the higher the score, the better the socioeconomic condition.

### 2.7 Body Mass Index (BMI)

Objective measurements of body weight (in kilograms) and height (in meters) were collected to calculate the body mass index (BMI = kg/m²). Body weight was assessed using a digital scale with a capacity of 180 kg and accuracy of 0.1 kg (WISO, model w912, Lot: 13A01, China), while height was measured using a portable stadiometer with a maximum capacity of 2.2 m and an accuracy of 0.1 cm. These measurements were collected with the participants barefoot and wearing light clothing. BMI was calculated through the division of body mass in kilograms by the height squared in meters and was analyzed as a continuous variable.

### 2.8 Disabling Chronic Diseases

Information about the presence of disabling chronic conditions was collected in the survey, in which participants were asked whether they had diabetes, myocardial infarction, osteoporosis, Alzheimer's, or Parkinson's disease diagnosed by a healthcare professional, how long ago the diagnosis was made, and whether they were using medications to treat the condition.

### 2.9 Statistical Analysis

Sample characteristics were reported using means and standard deviation. Logistic regression models were used to analyze the association of PA practice in life stages with QoL domains. The models were adjusted for sex, age, socioeconomic status, and the presence of disabling chronic conditions. The interaction effect of sex and socioeconomic status on the significant associations was explored by the creation of interaction terms (sex*PA engagement and SES*PA engagement), which were incorporated into specific dedicated models. For a collinearity check between variables, dummy variables were created for each category of independent variables included in the model, and it was analyzed if there were strong correlations between them (> 0.7). The goodness of fit for multivariate models was analyzed by the Hosmer and Lemeshow test *p* value. Statistical analysis was performed by IBM SPSS Statistical Package, with statistical significance set at two‐sided *p* < 0.05 level and the confidence interval at 95%.

## 3 Results

Table 1 demonstrates the characteristics of the sample, where 61.7% of the participants were female (Table 1). Considering PA, the approximate prevalence was 20% for engagement in PA during each life stage (childhood, adolescence, and adulthood). Regarding QoL, the social functioning domain had the highest score.

**Table 1:** Sample characteristics (*n* = 842).

	Mean (SD) or *N* (%)
Age (years)	56.5 (18.9)
Body mass index (kg/m²)	27.3 (5.7)
Childhood PA (%)	*N* = 182 (21.5)
Adolescence PA (%)	*N* = 180 (21.3)
Adulthood sports engagement (%)	*N* = 175 (20.7)
Physical activity (Baecke score ranging from 3 to 15)	11.7 (2.8)
Quality of life domain scores (ranging from 0 to 100)	
Physical functioning	73.6 (27.2)
Role physical	72.7 (42.3)
Bodily pain	65.1 (26.3)
General health	67.6 (20.8)
Vitality	69.8 (20.5)
Social functioning	81.6 (23.6)
Role emotional	79.8 (38.6)
Mental health	72.8 (19.6)
Chronic diseases	
Diabetes	*N* = 135 (16.0)
Myocardial infarction	*N *= 32 (3.8)
Osteoporosis	*N* = 58 (6.9)
Alzheimer's	*N* = 5 (0.6)
Parkinson's disease	*N* = 1 (0.1)
Socioeconomic level	
High	*N* = 177 (21.0)
Medium	*N *= 619 (73.3)
Low	*N* = 48 (5.7)

Abbreviations: PA = physical activity, SD = standard deviation.

The association of PA engagement in different life stages with QoL is presented in Table 2. Isolated models considering PA engagement in each life stage showed that PA engagement at childhood was associated with high QoL in domains of physical functioning (OR: 1.66 [95% CI: 1.12–2.45]), general health (OR: 1.90 [95% CI: 1.28–2.81]), social functioning (OR: 1.77 [95% CI: 1.20–2.60]), and mental health (OR: 1.99 [95% CI: 1.30–3.05]), whereas the PA engagement in adolescence showed no association with any QoL domain. Adults who were at top quartile of Baecke score were more likely to have high QoL in domains of physical functioning (OR: 1.71 [95% CI: 1.21–2.41]), general health (OR: 1.48 [95% CI: 1.06–2.07]), and role emotional (OR: 1.70 [95% CI: 1.11–2.60]). In addition, adults who reported to practice sports within the past 12 months showed higher odds of having high QoL in domains of physical functioning (OR: 2.07 [95% CI: 1.42–3.04]), bodily pain (OR: 1.50 [95% CI: 1.05–2.15]), general health (OR: 1.99 [95% CI: 1.39–2.83]), and vitality (OR: 1.52 [95% CI: 1.05–2.21]). When considering each life stage for PA engagement in the same statistical model, PA engagement at childhood remained significantly associated with high QoL in physical functioning (OR: 1.63 [95% CI: 1.09–2.42]), general health (OR: 1.89 [95% CI: 1.27–2.79]), social functioning (OR: 1.77 [95% CI: 1.20–2.60]) and mental health (OR: 1.99 [95% CI: 1.30–3.05]), whereas adulthood PA engagement remained associated with high physical functioning (OR: 1.70 [95% CI: 1.20–2.42]) and general health (OR: 1.47 [95% CI: 1.05–2.07]), and adulthood sports practice within the last 12 months remained associated with high QoL of physical functioning (OR: 1.96 [95% CI: 1.33–2.89]), bodily pain (OR: 1.52 [95% CI: 1.06–2.19]), and general health (OR: 1.86 [95% CI: 1.30–2.66]). The QoL domain of role physical showed no association with PA engagement in any life stage, as well as the domains of vitality and role emotional in the simultaneously adjusted models for each life stage PA engagement. No interaction effects were observed regarding the role of sex and socioeconomic status on the observed associations between PA engagement and QoL.

**Table 2:** Association of physical activity in different stages of life and the sum of physical activity in different stages of life with quality of life domains in adults (*n* = 842).

	Physical functioning	Role physical	Bodily pain	General health	Vitality	Social functioning	Role emotional	Mental health
	OR (95% CI)	OR (95% CI)	OR (95% CI)	OR (95% CI)	OR (95% CI)	OR (95% CI)	OR (95% CI)	OR (95% CI)
PA engagement (isolate life stage model)								
Childhood	**1.66 (1.12–2.45)**	1.34 (0.87**–**2.08)	1.03 (0.69**–**1.54)	**1.90 (1.28–2.81)**	1.48 (0.98**–**2.23)	**1.77 (1.20–2.60)**	0.78 (0.50**–**1.22)	**1.99 (1.30–3.05)**
H and L for model fit	0.94	> 0.99	0.94	0.97	0.37	0.41	0.21	0.76
Adolescence	1.28 (0.88**–**1.87)	0.91 (0.63**–**1.31)	0.83 (0.57**–**1.21)	1.06 (0.74**–**1.51)	1.33 (0.92**–**1.91)	1.02 (0.73**–**1.43)	0.89 (0.60**–**1.33)	1.07 (0.73**–**1.58)
H and L for model fit	0.90	0.85	0.67	0.16	0.58	0.64	0.52	0.92
Adulthood	**1.71 (1.21–2.41)**	1.12 (0.78**–**1.60)	1.30 (0.93**–**1.83)	**1.48 (1.06–2.07)**	1.26 (0.89**–**1.78)	0.97 (0.70**–**1.34)	**1.70 (1.11–2.60)**	0.98 (0.68**–**1.43)
H and L for model fit	0.68	0.77	0.91	0.44	0.69	0.41	0.07	0.49
Adulthood (sports only)	**2.07 (1.42–3.04)**	1.11 (0.75**–**1.65)	**1.50 (1.05–2.15)**	**1.99 (1.39–2.83)**	**1.52 (1.05–2.21)**	1.24 (0.87**–**1.75)	0.76 (0.50**–**1.14)	1.06 (0.71**–**1.58)
H and L for model fit	0.78	0.91	0.27	0.65	0.66	0.20	0.36	0.72
PA engagement (simultaneous life stage model)								
Childhood	**1.63 (1.09–2.42)**	1.34 (0.87**–**2.08)	1.02 (0.69**–**1.53)	**1.89 (1.27–2.79)**	1.47 (0.97**–**2.21)	**1.77 (1.20–2.60)**	0.78 (0.49**–**1.22)	**1.99 (1.30–3.05)**
Adolescence	1.29 (0.88**–**1.89)	0.91 (0.64**–**1.32)	0.84 (0.58**–**1.22)	1.06 (0.73**–**1.52)	1.34 (0.93**–**1.92)	1.01 (0.72**–**1.42)	0.91 (0.61**–**1.36)	1.07 (0.73**–**1.58)
Adulthood	**1.70 (1.20–2.42)**	1.11 (0.77**–**1.59)	1.30 (0.93**–**1.82)	**1.47 (1.05–2.07)**	1.26 (0.89**–**1.79)	0.96 (0.69**–**1.33)	1.71 (1.12**–**2.61)	0.97 (0.67**–**1.41)
H and L for model fit	0.44	0.76	0.76	0.83	0.28	0.69	0.73	0.83
Childhood	**1.50 (1.01–2.24)**	1.33 (0.85**–**2.06)	0.96 (0.64**–**1.44)	**1.74 (1.17–2.59)**	1.39 (0.92**–**2.11)	**1.73 (1.17–2.56)**	0.82 (0.52**–**1.28)	**2.01 (1.30–3.08)**
Adolescence	1.24 (0.85**–**1.83)	0.91 (0.63**–**1.31)	0.82 (0.56**–**1.19)	1.02 (0.71**–**1.48)	1.31 (0.91**–**1.89)	1.01 (0.72**–**1.42)	0.90 (0.61**–**1.35)	1.07 (0.73**–**1.58)
Adulthood (sports only)	**1.96 (1.33–2.89)**	1.07 (0.72**–**1.60)	**1.52 (1.06–2.19)**	**1.86 (1.30–2.66)**	1.44 (0.99**–**2.10)	1.15 (0.81**–**1.64)	0.78 (0.52**–**1.18)	0.96 (0.64**–**1.44)
H and L for model fit	0.17	0.53	0.40	0.46	0.01	0.48	0.61	0.94

*Reference group = Analysis comparing with participants who reported no PA engagement in any life stage. PA = physical activity; Adulthood PA = 4th quartile of Baecke score; H and L = Hosmer and Lemeshow test *p* value. Analysis adjusted by sex, age, socioeconomic level, and disabling chronic diseases (diabetes, myocardial infarction, osteoporosis, Alzheimer's, and Parkinson's). Bold values statistically significant.

Table 3 presents specific combinations and clustering of life stages with PA engagement associated with QoL domains. Specific combinations of two life stages with PA engagement were associated with high QoL in domains of physical functioning (childhood + adolescence OR: 3.34 [95% CI: 1.61–6.96]; childhood + adulthood OR: 3.39 [95% CI: 1.80–6.39]; adolescence + adulthood OR: 2.82 [95% CI: 1.39–5.71]; childhood + adulthood sports OR: 2.92 [95% CI: 1.59–5.37]; and adolescence + adulthood sports OR: 2.96 [95% CI: 1.46–6.02]), bodily pain (childhood + adulthood sports OR: 1.50 [95% CI: 1.06–2.12]), general health (childhood + adulthood OR: 2.88 [95% CI: 1.59–5.21]; childhood + adulthood sports OR: 2.80 [95% CI: 1.58–4.96]; adolescence + adulthood sports OR: 2.71 [95% CI: 1.41–5.20]), and vitality (childhood + adolescence OR: 2.63 [95% CI: 1.32–5.27]; childhood + adulthood OR: 2.08 [95% CI: 1.13–3.82]; adolescence + adulthood OR: 2.40 [95% CI: 1.23–4.69]). No association of a specific combination of two life stages of PA engagement was observed in QoL domains of role physical, social functioning, role emotional, and mental health.

**Table 3:** Association of physical activity in different stages of life and sum of physical activity in different stages of life with quality of life domains in adults (*n* = 842).

	Physical functioning	Role physical	Bodily pain	General health	Vitality	Social functioning	Role emotional	Mental health
	OR (95% CI)	OR (95% CI)	OR (95% CI)	OR (95% CI)	OR (95% CI)	OR (95% CI)	OR (95% CI)	OR (95% CI)
PA engagement (specific life stage combination)								
Childhood + adolescence	**3.34 (1.61–6.96)**	0.95 (0.45**–**2.01)	0.87 (0.41**–**1.82)	1.74 (0.87**–**3.46)	**2.63 (1.32–5.27)**	1.71 (0.87**–**3.38)	0.89 (0.41**–**1.93)	1.55 (0.71**–**3.37)
H and L for model fit	0.64	0.24	0.32	0.86	0.39	0.43	> 0.99	0.65
Childhood + adulthood	**3.39 (1.80–6.39)**	1.40 (0.69**–**2.81)	1.08 (0.58**–**2.01)	**2.88 (1.59–5.21)**	**2.08 (1.13–3.82)**	1.14 (0.64**–**2.06)	1.48 (0.70**–**3.12)	1.62 (0.84**–**3.11)
H and L for model fit	0.06	0.97	0.44	0.77	0.50	0.45	0.48	0.66
Adolescence + adulthood	**2.82 (1.39–5.71)**	1.09 (0.51**–**2.31)	1.03 (0.51**–**2.08)	1.90 (0.98**–**3.69)	**2.40 (1.23–4.69)**	1.13 (0.58**–**2.19)	2.20 (0.82**–**5.87)	1.39 (0.68**–**2.84)
H and L for model fit	0.80	0.88	0.27	0.61	0.73	0.66	0.59	0.30
Childhood + adulthood (sports only)	**2.92 (1.59–5.37)**	0.96 (0.67**–**1.38)	**1.50 (1.06–2.12)**	**2.80 (1.58–4.96)**	1.75 (0.96**–**3.19)	1.54 (0.87**–**2.71)	0.61 (0.32**–**1.17)	1.76 (0.95**–**3.29)
H and L for model fit	0.73	0.61	0.08	0.63	0.51	0.68	0.29	0.08
Adolescence + adulthood (sports only)	**2.96 (1.46–6.02)**	1.13 (0.53**–**2.40)	1.55 (0.80**–**3.01)	**2.71 (1.41–5.20)**	1.90 (0.97**–**3.75)	1.53 (0.79**–**2.97)	1.02 (0.45**–**2.33)	1.26 (0.61**–**2.60)
H and L for model fit	0.42	0.64	0.81	0.21	0.13	0.45	0.49	0.54
Clustering PA engagement^a^								
1. Life stage	1.18 (0.84**–**1.68)	1.15 (0.83**–**1.59)	1.17 (0.85**–**1.62)	1.34 (0.96**–**1.87)	1.12 (0.79**–**1.57)	1.33 (0.98**–**1.81)	1.03 (0.71**–**1.48)	**1.42 (1.01–2.01)**
2. Life stages	**2.49 (1.47–4.24)**	1.12 (0.64**–**1.98)	1.09 (0.64**–**1.86)	**2.09 (1.25–3.51)**	1.66 (0.97**–**2.83)	1.11 (0.67**–**1.84)	0.96 (0.53**–**1.75)	1.11 (0.61**–**2.02)
3. Life stages	**5.53 (1.70–17.99)**	1.27 (0.40**–**4.05)	0.92 (0.31**–**2.70)	**2.79 (1.05–7.40)**	**4.16 (1.54–11.26)**	1.85 (0.68**–**4.87)	2.81 (0.61**–**12.89)	**2.79 (1.01–7.73)**
H and L for model fit	0.89	0.64	0.12	0.92	0.72	0.41	0.32	0.46
1. Life stage (sports only)	1.32 (0.93**–**1.88)	0.97 (0.69**–**1.35)	1.11 (0.80**–**1.54)	**1.46 (1.04–2.04)**	1.36 (0.96**–**1.91)	1.24 (0.91**–**1‐69)	0.70 (0.48**–**1.01)	1.41 (0.99**–**1.99)
2. Life stages (sports only)	**2.38 (1.42–3.97)**	1.22 (0.70**–**2.12)	1.16 (0.70**–**1.92)	**2.42 (1.48–3.95)**	**1.99 (1.20–3.29)**	**1.67 (1.03–2.72)**	0.74 (0.41**–**1.32)	1.32 (0.76**–**2.28)
3. Life stages (sports only)	**5.57 (1.70–18.15)**	1.59 (0.44**–**5.73)	1.21 (0.43**–**3.39)	**2.83 (1.07–7.49)**	2.42 (0.88**–**6.65)	1.53 (0.57**–**4.08)	0.69 (0.30**–**2.10)	2.32 (0.81**–**6.65)
H and L for model fit	0.09	0.80	0.55	0.12	0.09	0.74	0.63	0.36

a Reference group = Participants who reported no PA engagement in any life stage. PA = physical activity; Adulthood PA = 4th quartile of Baecke score; H and L = Hosmer and Lemeshow test *p* value. Analysis adjusted by sex, age, socioeconomic level, and disabling chronic diseases (diabetes, myocardial infarction, osteoporosis, Alzheimer's, and Parkinson's). Bold values statistically significant.

The clustering of life stages with PA engagement showed an incremental association for high physical functioning (two life stages OR: 2.49; three life stages OR: 5.53), general health status (two life stages OR: 2.09; three life stages OR: 2.79), and mental health (one life stage OR: 1.42; three life stages OR: 2.79). When considering the adulthood sports instead of adulthood PA (top quartile of Baecke score), the incremental association for high physical functioning and general health remained significant, while the previous association with mental health became non‐significant. No association was observed regarding the clustering of lifelong PA engagement with QoL domains of role physical, bodily pain, social functioning, and role emotional.

## 4 Discussion

This study analyzed the association of lifespan PA practice with QoL domains. Our results indicated that high QoL domains were associated with adults who practiced PA during childhood (physical functioning, general health, social functioning, and mental health) and during adulthood (physical functioning, bodily pain, general health, vitality, and role emotional), whereas the PA engagement during adolescence showed no isolated association with QoL. Any dual combination of the three life stages with PA engagement was associated with high QoL, especially in domains of physical functioning, general health, and vitality. The clustering of life stages showed incremental association with high QoL in domains of physical functioning, general health, and mental health, when compared to no lifelong PA engagement.

Adults who practiced PA during their childhood and adolescence and those who are highly active have about three times more odds of having high QoL in domains of physical functioning, corroborating with previous studies in the literature [33, 34]. The lifelong active lifestyle has been associated with greater physical fitness [35], whereas sedentary children and adolescents are less likely to become active in adulthood [36]. High scores in the physical functioning domain may be a result of the benefits of physical activity acting as a protective factor against musculoskeletal problems [37, 38]. PA practice has been widely reported in the literature as a feasible therapeutic form for people experiencing pain conditions, considering the beneficial effects of improving cognitive and physical functions [39]. The present study observed that engagement in sports in adulthood was associated with 42% greater odds of having high QoL in domain of bodily pain.

Adults who had PA engagement in both childhood and adulthood were threefold more likely to have high general health. PA may positively influence the QoL, improving general health regarding its physical and mental aspects. One explanation is that the PA practice contributes to the release of well‐being hormones, such as endorphins and serotonin [40]. In addition, PA engagement during the stages of life helps to prevent several chronic diseases, which are the main ones responsible for situations of morbidity, such as obesity, hypertension, and dyslipidemia [41].

The PA engagement in childhood was associated with high social functioning and mental health in the present study sample. PA practice during daily life prevents mental issues and promotes health benefits such as better social insertion and convivence, a reduction of isolation feeling, improvement of people's commitment and cooperation in daily life activities, increase of vigor and self‐esteem, and, hence, a reduction of their inactivity. Regarding the emotional role, our study observed that adults at the top quartile of current physical activity were 70% more likely to have a high QoL score in this domain. A longitudinal investigation showed that depression was related to greater sedentary behavior, increasing the risk of depression in childhood, adolescence, and adulthood [42]. Regarding physiological and biochemical mechanisms, PA releases a wide range of neurotransmitters, which can have antidepressant effects [43]. In addition, the regular practice of exercise improves neural functions and structure, which may increase cognitive functions and prevent the negative effects of anxiety and depression [44].

The specific combinations of life stages with PA engagement showed different associations with QoL domains. The PA engagement in childhood + adulthood was associated with high general health, indicating that maintaining or resuming physical activity in adulthood after being engaged in PA in childhood may enhance general health benefits. In addition, any dual combination was associated with high physical functioning and high vitality, suggesting that PA engagement contributes to the preservation of functional capacity, energy, and disposition levels throughout life, regardless of when it was performed. Although studies investigating lifelong PA engagement with QoL are still incipient, previous research has shown cumulative PA effects on physical performance, strength, and cognitive function in later life [45, 46].

The QoL domain of role physical showed no association with lifelong PA engagement in the present study. This lack of association may be due to external factors which can minimize the impact of PA and directly influence the QoL, such as stress levels, inadequate sleep, eating habits, and medication use. Additionally, sample characteristics like age group, occupational conditions, and chronic diseases may impact the results, and different intensities, frequency, and continuity of lifelong PA as well.

This study has some limitations, such as the lack of recording the intensity and amount of PA practiced during childhood and adolescence (which might lead to overestimation of PA practiced). Nevertheless, the retrospective analysis of PA during childhood and adolescence can be susceptible to memory bias. The use of a questionnaire to assess the current PA is another limitation, which relies on the participant's report and perception, which can be susceptible to biases of memory and classification of intensity. However, the Baecke questionnaire seems to be a reliable and valid measurement of habitual PA in adults [31]. The larger sample size of women compared to men may also influence the results. Another point to be considered as a limitation is the risk of reverse causality when exploring current PA (since impaired QoL may affect the practice of PA). Besides having important limitations, it should be highlighted that the sample size, the randomized sampling process, and adjustment of the analysis by confounding factors as strengths of the study.

As a suggestion for future studies, we highlight the objective measurement of PA when possible. One possibility would be to measure, in addition to assessing previous PA, the intensities of current PA. This would allow us to verify whether participants who were active in childhood and adolescence with higher intensities of habitual PA would have a greater chance of having better QoL.

As practical applications of this study, we highlight the importance of policies that implement increased health promotion actions in different age groups. PA awareness programs in schools to include children and adolescents, as well as creating environments to encourage PA practice in the adult population, could be considered.

## 5 Conclusion

The PA practice in childhood and adolescence was associated with high QoL in adulthood when compared to people who did not practice PA previously. Participants engaged in PA in all stages of life (childhood, adolescence, and adulthood) were more likely to have high QoL in domains of physical functioning, role physical, general health, social functioning, emotional role, and mental health. Health promotion actions that include the practice of PA from childhood and adolescence (the school environment can be a key element), as well as policies to increase/maintain PA in adult life, need to be encouraged to maintain high QoL.

## Author Contributions

**Ewerton P. Antunes:** conceptualization, methodology, writing – original draft. **Wagner L. Prado:** conceptualization, writing – review and editing, data curation, validation. **Daniel Canhin:** conceptualization, writing – original draft. **Claudiele C. M. Silva:** writing – review and editing. **Luis Alberto Gobbo:** writing – review and editing. **Gerson Ferrari:** writing – review and editing. **Jorge Mota:** writing – review and editing, visualization. **William R. Tebar:** methodology, data curation, project administration, formal analysis, writing – review and editing. **Diego G. D. Christofaro:** conceptualization, investigation, formal analysis, project administration, supervision, data curation, writing – review and editing, visualization, methodology, validation.

## Conflicts of Interest

The authors declare no conflicts of interest.

## Transparency Statement

The corresponding author, Wagner L. Prado, affirms that this manuscript is an honest, accurate, and transparent account of the study being reported; that no important aspects of the study have been omitted; and that any discrepancies from the study as planned (and, if relevant, registered) have been explained.

## Data Availability

The data that support the findings of this study are available from the corresponding author upon reasonable request.
